# The Impact of Glacial Disturbance History Upon the Genetic Diversity of *Unio crassus* and *Unio nanus* in Europe and Implications for Conservation

**DOI:** 10.1002/ece3.72113

**Published:** 2025-09-06

**Authors:** Sarah Egg, Manuel Lopes‐Lima, Helmut Bayerl, Elsa Froufe, Bernhard C. Stoeckle, Ralph Kuehn, Juergen Geist

**Affiliations:** ^1^ Aquatic Systems Biology Unit TUM School of Life Sciences, Technical University of Munich Freising Germany; ^2^ Molecular Zoology Unit TUM School of Life Sciences, Technical University of Munich Freising Germany; ^3^ CIBIO, Centro de Investigação Em Biodiversidade e Recursos Genéticos, InBIO Laboratório Associado Campus de Vairão, Universidade do Porto Vairão Portugal; ^4^ BIOPOLIS Program in Genomics, Biodiversity and Land Planning, CIBIO Campus de Vairão Vairão Portugal; ^5^ CIIMAR/CIMAR–Interdisciplinary Centre of Marine and Environmental Research, University of Porto Terminal de Cruzeiros Do Porto de Leixões, Av. General Norton de Matos s/n Matosinhos Portugal; ^6^ ICBAS–School of Medicine and Biomedical Sciences, U. Porto University of Porto, Laboratory of Cytogenetics Porto Portugal

## Abstract

Historically, the thick‐shelled river mussel ( *Unio crassus* agg. complex) was considered a single, widespread species across Europe. However, recent phylogenetic taxonomic revisions have delineated 12 species from this complex, including *Unio crassus* (s. str. Philipsson in Retzius, 1788) and *Unio nanus* (Lamarck, 1819 stat. rev.), which exhibit substantial range overlap and broad European distributions. Understanding their fine‐scale genetic diversity, population structure, and potential for recent or ancient hybridization is critical for effective conservation planning. This study investigated the genetic diversity and structure of *U. crassus* and *U. nanus* across Europe, examining the influence of glacial disturbance history and host‐fish associations. Using mitochondrial (COI) and nuclear (microsatellite) markers on 60 populations, we revealed a discordance between mitochondrial and nuclear structuring, suggesting ancient introgression. Crucially, no evidence of recent hybridization was detected between *U. crassus* and *U. nanus* . We found significantly higher nuclear genetic diversity in *U. crassus* compared to *U. nanus* . Our findings indicate an older Black Sea–Caspian Sea divergence and ancient introgression between *U. nanus* and *U. crassus* , as well as distinct postglacial colonization routes: a Western route for *U. nanus* and an Eastern route for *U. crassus* , converging in a secondary contact zone. Our results highlight the strong influence of host‐fish associations and glacial history in shaping the genetic patterns of these mussels, underscoring the need to incorporate intraspecific genetic diversity into conservation strategies. As shell morphology proved unreliable for species identification, we recommend DNA barcoding for reliable species recognition and suggest further research into host‐fish preferences to improve conservation efforts.

## Graphical abstract

This paper presents novel findings on the genetic diversity of the recently described thick‐shelled river mussel complex, combining nuclear, mitochondrial, and morphometric markers in relation to European host‐fish colonization patterns. In particular, we identified currently unknown contact zones across their European distribution and found pronounced differences in the genetic diversity among the different species within the *U. crassus* complex.

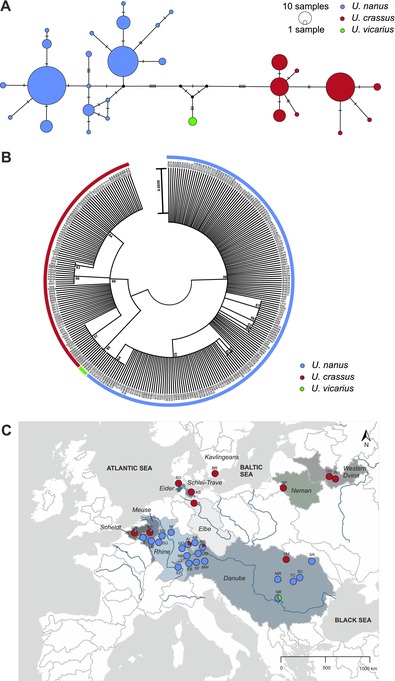

## 1 Introduction

The conservation of intraspecific genetic diversity is increasingly recognized as crucial for species survival and is a key objective of international biodiversity agreements, such as the Kunming‐Montreal Targets for 2030 (Target 4) of the United Nations Convention on Biological Diversity (CBD) or the European Biodiversity Strategy for 2030. Assessing genetic diversity and prioritizing conservation units within threatened species is critical for effective conservation management (Alves et al. 2023; Geist 2010; Ottewell and Byrne 2022). Genetic diversity is also a valuable indicator of a population's evolutionary potential, particularly when the genetic basis of adaptive traits is poorly understood (Willi et al. 2022). Furthermore, quantitative metrics of genetic diversity serve as proxies for extinction risk, as they are positively correlated with population fitness and size, and thus, population viability (Frankham 2012; McLaughlin et al. 2025). Loss of genetic diversity within small populations reduces adaptive potential and increases extinction risk (Frankham et al. 2017). Despite the recognized importance of genetic data, its systematic integration into practical conservation management and species threat assessments, such as the IUCN Red List, remains limited (McLaughlin et al. 2025).

The *Unio crassus* agg. complex, a group of threatened European freshwater mussels that is legally protected under the EU Habitats Directive (Annex II and IV), exemplifies the significant challenges in conserving widespread species. A previous study on population genetic studies of *U. crassus* (Philipsson 1788), using microsatellite markers, revealed high genetic differentiation among populations in Germany and Sweden, suggesting a strong influence of Pleistocene glaciations on its spatial genetic structure (Feind et al. 2018). However, this study did not fully consider the demographic history of host fish or phylogenetic relationships between populations. Recent molecular investigations based on phylogenetic analyses (whole mitogenomes, 800 genes, and COI) have fundamentally re‐evaluated the *U. crassus* complex, revealing a complex of at least 12 distinct species, many of which are morphologically cryptic (Lopes‐Lima, Geist, et al. 2024). Among these newly recognized species, *Unio crassus* (s. str. Philipsson in Retzius, 1788), *Unio nanus* (Lamarck, 1819 stat. rev.), and *Unio vicarius* (Westerlund in Westerlund and Blanc, 1879 stat. rev.) exhibit substantial range overlap and broad distributions across the temperate regions of the European continent, complicating species delineation and conservation planning. The extent of genetic mixing, including hybridization and its impact on the long‐term viability of these three species, remains unclear. The underlying causes of hybridization are complex and deep, and considerations of their complexity can help to reveal evolutionary pathways of species (Bennett 1997; Hewitt 2011).

While *U. crassus* was historically thought to be widespread across much of Europe (excluding Britain, Italy, and the Iberian Peninsula), the recent re‐evaluation has likely refined its actual distribution (Lopes‐Lima, Prié, et al. 2024). It is endemic to Europe, found in most Atlantic and Baltic river basins from France to Russia, and in Eastern European river basins draining into the Caspian and Black Seas. *U. nanus* is now recognized in Europe, with its core distribution primarily in the Rhône, Rhine, and Danube basins (Lopes‐Lima and Prié 2024). Its range includes countries like France, Germany, Austria, Switzerland, and others in Central and Eastern Europe, often overlapping with the broader, but now more narrowly defined, distribution of *U. crassus* . The observed sympatry and close phylogenetic proximity of *U. crassus* and *U. nanus* underscore the critical need to characterize the extent and evolutionary implications of potential hybridization and introgression for conservation (Lopes‐Lima, Geist, et al. 2024). Both *U. crassus* and *U. nanus* face severe conservation pressure, evidenced by population declines exceeding 50% over the past 60 years across their broad European distribution (Lopes‐Lima and Prié 2024; Lopes‐Lima, Prié, et al. 2024). These declines are particularly acute in certain regions, with reported population reductions of up to 90% in Germany and local extirpations, such as in the Netherlands (Lopes‐Lima, Prié, et al. 2024; Nienhuis 2012; Zettler and Jueg 2007). Consequently, *U. crassus* and *U. nanus* are categorized as ‘Endangered’ on the IUCN Red List, primarily due to habitat degradation, pollution, and the decrease of host‐fish populations (Lopes‐Lima and Prié 2024, Lopes‐Lima, Prié, et al. 2024). *U. vicarius* is primarily distributed in the Balkan region, extending eastwards to Romania and Greece (Lopes‐Lima, Prié, and Karaouzas 2024). It co‐occurs with *U. crassus* and with *U. nanus* , specifically in the Northern Balkan regions, for example in the Lower and Middle Danube in Romania. This species is categorized as ‘Vulnerable’ on the IUCN Red List, and the current population trend is decreasing. Substantial conservation efforts, including EU LIFE projects with a financial commitment of approximately €120 million, reflect the urgency of protecting these species (European Commission LIFE Public Database 2025; Geist et al. 2023; Lopes‐Lima, Geist, et al. 2024; Zając, Florek, et al. 2018).

In general, unionid mussels possess a life cycle characterized by a parasitic larval stage (glochidia) that necessitates temporary attachment to freshwater fishes for development and dispersal, often displaying significant host specificity (Barnhart et al. 2008; Bauer 2001). The distribution and genetic structure of unionid mussels are therefore strongly linked to the ecology and evolutionary history of their host fish. For *U. crassus* and *U. nanus* , regional heterogeneity in host‐fish utilization and evidence of local adaptation are frequently documented (Lamand et al. 2016; Lopes‐Lima et al. 2017; Schneider et al. 2017; Stoeckl et al. 2015; Taeubert et al. 2012). This dependency underscores the critical role of fish in the biogeography of unionid mussels, as evidenced by the congruence between mussel genetic vicariance and host‐fish demographic history (Elderkin et al. 2007; Johnson et al. 2024).

To address these knowledge gaps and inform conservation strategies, this study employs a combined approach using mitochondrial and nuclear genetic markers to investigate the genetic diversity and genetic structure of *U. crassus* and *U. nanus* across their European distribution. Specifically, we aim to:

- Assess the levels of genetic diversity and variation within and among populations of *U. crassus* and *U. nanus* across its European distribution, employing a multilocus approach to elucidate population genetic parameters and phylogeographic patterns.
- Delineate the genetic structure of populations, with a specific focus on the differentiation and phylogenetic relationships between *U. crassus* and *U. nanus* , utilizing population genetic and phylogeographic methodologies to infer evolutionary species and population connectivity.
- Test for potential hybridization and/or introgression within sympatric populations (between *U. crassus* x *U. nanus* and *U. vicarius* x *U. nanus* ),
- Elucidate the relationship between genetic constitution and shell morphology in *U. crassus* and *U. nanus* across Europe, investigating the genetic basis of phenotypic variation through morphometric and molecular analyses,
- Reconstruct the postglacial colonization history of *U. crassus* and *U. nanus across* Europe in relation to the phylogeography of primary host‐fish species, integrating phylogeographic data from both mussels and their host species to infer dispersal routes and colonization events.

## 2 Materials and Methods

### 2.1 Sample Collection and DNA Extraction

Sample collection was conducted between 2015 and 2018. Sample material was obtained using nonlethal methods, employing either hemolymph extraction (Geist and Kuehn 2005) or tissue sampling (Naimo et al. 1998). DNA was extracted from tissue samples using a phenol–chloroform–isopropanol extraction protocol (Hogan et al. 1986). For hemolymph samples, DNA extraction was performed with the NucleoSpin Tissue Kit (Macherey–Nagel) according to (Geist and Kuehn 2005). To broaden the scope of the analysis, genotypes from a previous population genetic study of *U. crassus* (Feind et al. 2018) were included as a reference. In total, the present study encompassed 1531 specimens from 60 thick‐shelled river mussel populations across 10 major drainage systems in Europe. Detailed sampling and population information is provided in Table S1.

### 2.2 mtDNA Analysis

Mitochondrial DNA (mtDNA) analysis was conducted on a subset of 300 specimens, selected from the total pool of 1531 individuals (Table 1, Table S1). To specifically address patterns within major European drainage systems, a subset comprising 300 individuals from 30 representative populations from all major drainage systems, with a balanced sampling of 10 individuals per population, was selected for further analysis (Table S1). A fragment of the female (*F*‐type) cytochrome c oxidase subunit I (COI) gene was utilized for this analysis (Froufe et al. 2014), with 286 sequences obtained from a previous phylogenetic study of the *Unio crassus* complex (Lopes‐Lima, Prié, et al. 2024). Additional specimens (*n* = 14) were analyzed using the *Unio*‐specific primer pair Unio COI‐F: 5′‐GTCTGGGTTAATTGGRTTRG‐3′ and Unio COI‐R: 5′ CCAGCTAARACAGGYAAAGCRG‐3′ due to high loads of bacteria in the hemolymph samples. PCR was carried out with the following PCR profile: initial denaturation at 94°C for 3 min, 35 cycles of 94°C for 30 s, 58°C for 90 s, and 72°C for 30 s, before final extension at 72°C for 3 min and cooling to 10°C. Sequences were aligned and trimmed to a standardized length of 442 bp using ClustalW in MEGA11 software (Tamura et al. 2021). The best‐fit model of nucleotide substitution was then determined and subsequently employed to construct a maximum likelihood (ML) phylogenetic tree with 1000 bootstrap replicates. To assess genetic diversity within and among populations, DnaSP v6.12 (Rozas et al. 2017) was used to calculate the following parameters: number of haplotypes (*N*_hap_), number of polymorphic sites (*S*), haplotype diversity (Hd), nucleotide diversity (*π*), and Watterson's estimator of theta (*ϴ*) (Watterson 1975). Finally, a median‐joining haplotype network was generated using PopART v1.7 (Leigh and Bryant 2015) to visualize relationships among haplotypes and species.

**TABLE 1:** Genetic diversity indices based on the COI sequences of 30 thick‐shelled river mussel populations with population code (POPID), species, sample size, number of haplotypes (*N*_hap_), number of variable sites (*S*), haplotype diversity (Hd), nucleotide diversity (*π*), and Watterson's estimator of theta (*ϴ*).

POPID	Species	*N*	*N*_hap_	*S*	Hd	*π*	*ϴ*
GH	*U. crassus* / *U. nanus*	10	4	18	0.533	0.013	0.015
HE	*U. nanus*	10	2	3	0.200	0.001	0.003
ME	*U. crassus* / *U. nanus*	10	2	15	0.356	0.013	0.013
LE	*U. nanus*	10	3	4	0.644	0.004	0.003
SU	*U. nanus*	10	2	3	0.533	0.004	0.003
XO	*U. nanus*	10	5	8	0.800	0.005	0.007
NI	*U. nanus*	10	5	8	0.800	0.006	0.007
GG	*U. nanus*	10	3	4	0.600	0.004	0.003
AI	*U. crassus* / *U. nanus*	10	4	17	0.800	0.018	0.014
AB	*U. nanus*	10	1				
SZ	*U. nanus*	10	1				
NB	*U. nanus*	10	3	5	0.711	0.005	0.004
EB	*U. nanus*	10	2	3	0.200	0.001	0.003
SF	*U. nanus*	10	1				
MW	*U. nanus*	10	2	4	0.200	0.002	0.003
DM	*U. nanus*	10	4	7	0.644	0.005	0.006
RG	*U. crassus* / *U. nanus*	10	5	18	0.844	0.016	0.015
MR	*U. nanus*	10	4	7	0.644	0.004	0.006
NR	*U. nanus* / *U. vicarius*	10	3	14	0.644	0.017	0.012
SM	*U. crassus*	10	3	4	0.378	0.002	0.004
TC	*U. nanus*	10	1				
SC	*U. nanus*	10	3	5	0.644	0.006	0.004
SR	*U. nanus*	10	4	4	0.778	0.005	0.004
IL	*U. crassus*	10	2	1	0.200	0.000	0.001
AS	*U. crassus*	10	1				
BO	*U. crassus*	10	1				
BR	*U. crassus*	10	1				
SP	*U. crassus*	10	3	4	0.600	0.004	0.003
DG	*U. crassus*	10	4	5	0.533	0.002	0.004
IN	*U. crassus*	10	5	6	0.844	0.006	0.005
Overall		300	29	38	0.840	0.018	0.016

### 2.3 Microsatellite Analysis

Microsatellite genotyping was performed on 1531 specimens using a panel of eight established loci (Uc5, Uc15, Uc16, Uc19, Uc25, Uc39, Uc69, and Uc77) (Feind et al. 2018; Sell et al. 2013). PCR amplification and subsequent fragment length analysis followed the protocols detailed in Feind et al. (2018). Microsatellite raw data was managed using the EXCEL MICROSATELLITE TOOLKIT 3.1 (Park 2008) for initial input file preparation. The microsatellite dataset was tested for the presence of null alleles, large allelic dropout, and stuttering with MICROCHECKER 2.2.3 (Van Oosterhout et al. 2004). To quantify the impact of high null allele frequencies on *F*_ST_ estimates, FREENA (Chapuis and Estoup 2007) was used by comparing *F*_ST_ estimates before and after the correction of null alleles applying the ENA method. Alleles were considered private (*A*_P_) if occurring uniquely in a population and showing an allelic frequency > 5% to exclude loci outliers. Genetic diversity within populations was assessed by calculating the average allele number per locus (*A*), allelic richness (*A*_R_) as a standardized measure of the number of alleles corrected for the minimum sample size, expected and observed heterozygosities (*H*_E_, *H*_O_) using the R package ‘hierfstat.’ *F*_IS_ values estimating the degree of inbreeding within populations were calculated using GENEPOP 4.7.5 (Raymond and Rousset 2004). The probability of allelic coancestry in populations was assessed based on the *F*‐value from the software 2MOD (Ciofi et al. 1999) applying 100,000 iterations with the initial 10% of iterations discarded to avoid bias in parameter estimation due to starting conditions. Genetic diversity parameters, including measures of allelic richness, heterozygosity, and inbreeding coefficients, were compared among classified species using pairwise Mann–Whitney *U* tests (PAST 4.12b). *p*‐values were adjusted using a Bonferroni correction to account for multiple comparisons. This analysis aimed to identify significant variations in genetic diversity associated with species classification at the taxonomic level.

Evidence for recent genetic bottlenecks in populations was evaluated by testing for heterozygosity excess in the TPM model as recommended in BOTTLENECK 1.2.02 (Piry et al. 1999). Significance was assessed using the Wilcoxon signed‐rank test with 10,000 iterations, and the distribution of allele frequency classes was explored for deviation from the normal L‐shaped distribution. Genetic differentiation between populations was quantified by calculating estimates of pairwise *F*_ST_ values and global F‐statistics (Weir and Cockerham 1984) using ARLEQUIN 3.5 (Excoffier et al. 2005). Analysis of molecular variance (AMOVA, Excoffier et al. 1992) as implemented in ARLEQUIN 3.5 was executed to hierarchically evaluate the amount of genetic population structure. Variance components were extracted for the following hierarchical levels: (i) among species, (ii) among major drainage systems, (iii) among populations, (iv) among individuals within populations, and (v) within individuals. The Bayesian model‐based clustering method, STRUCTURE 2.3.4 (Pritchard et al. 2000), was executed to assess population genetic structure, to infer the number of genetic clusters (*K*), and to assign the probabilities of an individual to belong to each cluster. The number of clusters ranging from 1 to 20 was tested with 10 iterations for each *K*, applying 20,000 burn‐ins and 200,000 Markov chain Monte Carlo replicates in each run. The analyses were run without prior population information, assuming the ‘Admixture model’ and correlated allele frequencies. The selection of the final optimal number of clusters was based on ΔK, the rate of change in the log probability over all 10 iterations, as described in Evanno et al. (2005) using STRUCTURE HARVESTER (Earl and VonHoldt 2012). CLUMPP 1.1.2 (Jakobsson and Rosenberg 2007) was executed to determine the individual membership coefficients of replicated cluster analyses applying the Greedy algorithm with 10,000 random input orders. For comparative purposes, a second Bayesian clustering analysis was performed using NEWHYBRIDS 1.1 (Anderson and Thompson 2002) using the same parameters as employed in the STRUCTURE analysis. This analysis aimed to estimate the posterior probability of each individual belonging to one of six predefined categories: purebred parental types = Cluster 1 or Cluster 2, F1 hybrids = F1, F2 hybrids = F2, and the first two parental backcross hybrids = Bx1 or Bx2 using a threshold value of *q* = 0.75; individuals with < 0.75 remained unassigned (Neri et al. 2017). Averaged individual membership coefficients obtained from STRUCTURE 2.3.4 and NEWHYBRIDS 1.1 were then displayed with DISTRUCT 1.1 (Rosenberg 2004). Genetic structure was assessed by performing discriminant analysis of principal components (DAPC) (Jombart et al. 2010) using the R package ADEGENET, being less sensitive to unequal sampling size than Bayesian clustering approaches (Puechmaille 2016). Further, the DAPC method provides a model‐free assessment of between‐population genetic structures within negligible computational time, permitting the inference of complex patterns such as hierarchical clustering or clines. To describe the genetic similarity between individuals and populations, the result of the DAPC was visualized with a scatterplot where each principal component was recoded as intensities of a given color channel of the RGB system (Jombart 2008; Jombart et al. 2010). The RGB‐coded color plot obtained from the DAPC was then combined with a geographical map including the sampling locations of populations using the R packages MAPS and MAPDATA to illustrate the spatial genetic constitution between populations. To elucidate the postglacial colonization history of the thick‐shelled river mussel, we reconstructed potential dispersal pathways by tracing the distributions of two phylogenetically and ecologically distinct host‐fish species: the temperate‐adapted European chub (*Squalius cephalus*) and the cold‐adapted European bullhead ( *Cottus gobio* ). These species were specifically selected as proxies for distinct postglacial environmental conditions and associated colonization dynamics. Our rationale for focusing on *S. cephalus* and *C. gobio* , rather than other potential host fishes, rests on the following key considerations:

- Broad and partially overlapping distributions: Both species possess extensive geographical ranges across Europe (Kottelat and Freyhof 2007) that overlap significantly with the current distribution of *U. crassus* , *U. nanus* , and *U. vicarius*. This spatial congruence makes them plausible vectors for mussel dispersal during postglacial range expansion.
- Contrasting thermal ecologies: The fundamental difference in their thermal requirements—*S. cephalus* exhibiting a preference for warmer, temperate waters and *C. gobio* being adapted to colder conditions (Kottelat and Freyhof 2007)—allows us to hypothesize about the timing and directionality of mussel colonization events following glacial retreat and subsequent climate shifts. Tracing mussel genetic species associated with these distinct hosts could therefore provide insights into colonization routes favored during different postglacial climatic phases.
- Established host specificity: While the *U. crassus* complex is known to utilize multiple fish species as hosts for its larval stage (glochidia), both *S. cephalus* and *C. gobio* are frequently reported as primary hosts across a significant portion of the mussel's range (Ćmiel et al. 2018; Lamand et al. 2016; Taeubert et al. 2012; Schneider et al. 2017). However, these established host associations were typically determined without considering the species and, therefore, do not inherently differentiate the host utilization patterns of the closely related species *U. crassus* and *U. nanus* .

### 2.4 Shell Morphology

Shell shape variation among populations and species was assessed using elliptic fourier analysis (EFA), following the methodology outlined by Crampton (1995). Digital images of the left valve of 100 specimens from 10 populations (Table S1) were captured against a white background to maximize contrast. Shell outlines were extracted from these images using GIMP 2.10.32. Subsequently, 20 elliptic Fourier descriptors (EFDs) were generated for each shell using the Elliptic Fourier Descriptors plugin in ImageJ (Boudier and Tupper 2009). The first two EFDs, which do not convey shape information, were excluded from further analysis. A principal component analysis (PCA) was conducted on the remaining 18 EFDs to reduce dimensionality and visualize shape variation (PAST 4.12b). To test for significant differences in shell shape among populations and species, a linear discriminant analysis (LDA) with jack‐knifed prediction was performed (PAST 4.12b).

## 3 Results

### 3.1 mtDNA Analysis

**FIGURE 1:**
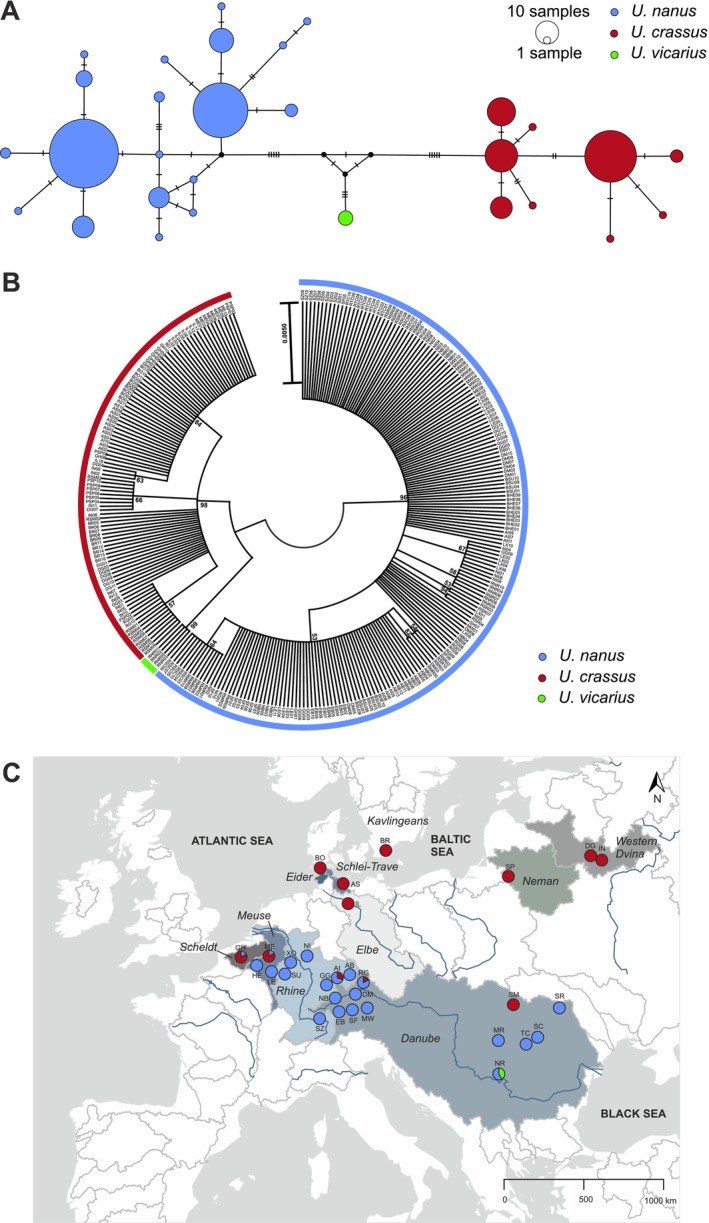
(A) Median‐joining haplotype network of 300 individuals obtained from the COI gene sequence analysis. The number of mutations is shown by hatch marks on the line. Colors correspond to species defined from median vectors representing a hypothesized ancestral sequence using maximum parsimony. (B) Maximum likelihood phylogenetic tree of 300 individuals obtained from the COI gene sequence analysis. Colors correspond to species derived from tree branches with bootstrap support values > 95%. (C) Phylogeographic distribution of species based on the COI analysis. Circles represent the location of populations; population codes (POPID) are according to Table S1.

Analysis of the mitochondrial cytochrome c oxidase subunit I (COI) gene revealed 29 distinct haplotypes, which were assigned to three distinct species according to (Lopes‐Lima, Geist, et al. 2024): *U. crassus* , *U. nanus* , and *U. vicarius* (Figure 1A). Model selection based on the BIC/AIC score in MEGA11 identified the Hasegawa‐Kishino‐Yano model with a discrete Gamma distribution (HKY + G) as the most appropriate model of nucleotide substitution. Phylogenetic reconstruction using maximum likelihood (ML) yielded a tree with robust support for the three designated species, as evidenced by high bootstrap values > 95% (Figure 1B). Among the sampled populations, 17 were identified as pure *U. nanus* , eight as pure *U. crassus* , four as admixed *U. crassus* × *U. nanus* , and one as admixed *U. nanus* × *U. vicarius* . The geographic distribution of *U. crassus* , *U. nanus* , and *U. vicarius* across Europe reveals distinct species differentiation and zones of potential admixture, as illustrated in Figure 1C. *U. crassus* is predominantly distributed across Northern Europe, encompassing the Baltic basins and parts of the North Sea basins. Conversely, *U. nanus* is primarily located in central and southern Europe, including the Danube River basin and extending into the Scheldt‐Meuse‐Rhine basins. *U. vicarius* appears to have a restricted distribution in the Balkan Danube basin, where it exhibits admixture with *U. nanus* . Notably, the Scheldt‐Meuse‐Rhine basins represent potential zones of admixture, with the presence of both *U. crassus* and *U. nanus* suggesting historical or ongoing gene flow between these species.

In total, 38 polymorphic sites (S) were detected, ranging from 1 to 18 sites per population (Table 1, Figure 3A). Sympatric *U. crassus* × *U. nanus* or *U. crassus* × *U. vicarius* populations exhibited a significantly higher number of polymorphic sites than pure *U. crassus* or *U. nanus* populations (Mann–Whitney *U*‐test, *p* = 0.01), while no significant difference in the number of polymorphic sites (S) was observed between *U. crassus* and *U. nanus* populations (Mann–Whitney *U*‐test, *p* = 1.00). Notably, several *U. crassus* populations from Northern Europe (e.g., AS, BO, BR) and *U. nanus* populations from the peripheral Danube region (e.g., SZ, SF, TC) displayed only one haplotype, indicating a strong historical founder effect. Haplotype gene diversity (Hd) ranged from 0.200 to 0.844 across populations, with no substantial differences observed among the three species (Mann–Whitney *U*‐test, *p* = 1.00). Nucleotide diversity (*π*) varied from 0.000 (IL population) to 0.018 (AI population). Although *U. crassus* populations generally exhibited lower *π* than sympatric populations, this difference was not statistically significant (Mann–Whitney *U*‐test, *p* = 0.06). Conversely, *U. nanus* populations showed significantly lower π compared to sympatric populations (Mann–Whitney *U*‐test, *p* = 0.01). Watterson's theta (θ) followed a similar pattern as *π*, with lower values observed in *U. crassus* and *U. nanus* populations relative to sympatric populations. The difference in θ between *U. nanus* and sympatric populations was statistically significant (Mann–Whitney *U*‐test, *p* < 0.01).

### 3.2 Microsatellite Analysis

Assessment of microsatellite data quality using MICROCHECKER 2.2.3 revealed no evidence of large allelic dropout or stuttering but indicated the potential presence of null alleles. To evaluate the impact of these putative null alleles on population genetic inferences, we compared *F*_ST_ estimates with and without correction for null alleles using FREENA. The observed differences were negligible (overall loci, ENA‐corrected *F*_ST_ = 0.108; uncorrected *F*_ST_ = 0.113). Given the minimal influence of null alleles on estimates of genetic differentiation, all loci were retained for subsequent analyses. Global *F*‐statistics, calculated across all loci and populations, demonstrated significant deviations from panmixia (*F*_IS_ = 0.111, *F*_ST_ = 0.112, *F*_IT_ = 0.210; *p* < 0.001 for all). Notably, the *F*_IT_ value, a measure of overall genetic differentiation, was two‐fold higher than the *F*_ST_ value and *F*_IS_ value, which quantify differentiation among subpopulations. This discrepancy suggests a hierarchical population structure, with substantial genetic divergence among local populations. AMOVA revealed that 78.6% of the genetic variation was found within individuals, while 11.3% of the genetic variation was explained among populations. 10.1% of the genetic variation was based on individuals within populations (Table S2). When examining the hierarchical partitioning of genetic variation with respect to major drainage systems, 4.0% was attributed to differences among drainage systems, while 8.6% was explained by variation among populations within drainage systems, and 87.4% resided within populations. Similarly, with respect to species, 6.2% of the genetic variation was attributed to differences among species, 10.5% to variation among populations within species, and 83.3% to variation within populations. These results suggest that while there is detectable genetic structure among populations and higher‐level groupings (drainage systems and species), genetic variation is predominantly found within individuals and populations.

**FIGURE 2:**
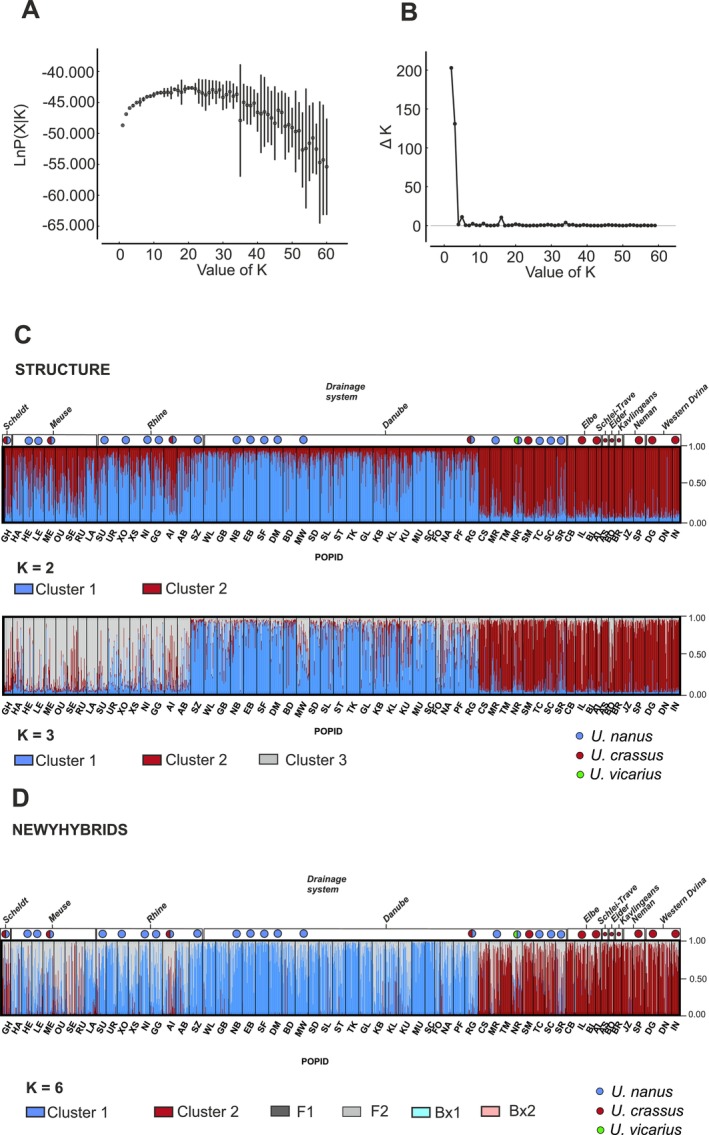
(A) Plot of mean log likelihood of *K* and variance per *K* value. (B) Plot of DeltaK (= Δ*K*) values derived from STRUCTURE Harvester analysis of 1531 individuals based on microsatellite data testing clusters ranging from 1 to 60 using 10 iterations for each *K*. (C) Bar plot of the STRUCTURE analysis for the most likely number of genetic clusters, *K* = 2 and *K* = 3, using 1531 individuals sorted by populations and drainage systems. Columns in the bar plot represent the assignment probability of each individual belonging to the given cluster. Dots on top of the bar plot indicate the species membership of populations based on the COI analysis. (D) Bar plot of the NEWHYBRIDS analysis using 1531 individuals classified to the six predefined genotype classes: Purebred parental types = Cluster 1 or Cluster 2, F1 hybrids = F1, F2 hybrids = F2, and the first two parental backcross hybrids = Bx1 or Bx2. Columns in the bar plot represent the assignment probability of each individual belonging to the given genotype class. Dots on top of the bar plot indicate the species membership of populations based on the COI analysis; population codes (POPID) are according to Table S1.

Analysis of population genetic structure using the software STRUCTURE 2.3.4 and subsequent evaluation with STRUCTURE HARVESTER revealed distinct clustering patterns within the dataset. Evaluation of Δ*K*, a measure of the rate of change in the log probability of the data between successive *K* values, indicated two prominent peaks corresponding to *K* = 2 (Δ*K* = 203) and *K* = 3 (Δ*K* = 131). These peaks suggest the presence of two or three genetically distinct clusters within the studied populations (Figure 2A,B). At *K* = 2, a clear division is observed between a cluster predominantly composed of Balkan and Northern populations, exhibiting high assignment probabilities (> 0.69 for Balkan and > 0.87 for Northern populations), and a second cluster largely consisting of Danubian populations with assignment probabilities ranging from 0.64 to 0.94 (Figure 2C). Populations from the Scheldt‐Meuse‐Rhine region display a mixed genetic composition at *K* = 2, indicating admixture between the two primary clusters. Increasing the number of clusters to *K* = 3 reveals further substructure. Notably, the Scheldt‐Meuse‐Rhine populations now form a distinct cluster, while the Balkan‐Northern and Danubian clusters remain largely consistent with the *K* = 2 analysis. Interestingly, the genetic clusters identified through STRUCTURE analysis do not fully align with the species classification inferred from phylogenetic reconstruction (Figure 1B). This discrepancy suggests that the observed genetic structure may be influenced by spatial factors, such as isolation by distance or local adaptation, rather than solely reflecting deep evolutionary divergence. Analysis with NEWHYBRIDS revealed a pattern of genetic differentiation consistent with the two‐cluster model identified in the STRUCTURE analysis (Figure 2D). Notably, NEWHYBRIDS detected no F1 hybrids (0%) or backcross hybrids (0%), but identified a substantial proportion of F2 hybrids (12%) within the dataset. This pattern suggests that hybridization between the two genetic clusters is not a recent phenomenon but rather represents historical introgression that has persisted over multiple generations.

DAPC analysis revealed a lack of distinct genetic clustering at the individual and population levels (Figure S1A), with high levels of within‐individual genetic variation (Figure S1B). However, spatial genetic patterns are evident along drainage systems. Specifically, populations within the Scheldt (GH), Meuse (HA, HE, ME, LE, SE, OU, LA, RU, SU), and Rhine (UR, XO; XS, NI, GG, AI, AB) basins exhibit relatively similar genetic compositions, indicative of genetic admixture. Likewise, populations from the Eider (BO), Schlei‐Trave (AS), Elbe (AL, BL, IL, CB), Kavlingeans (BR), Neman (JZ, SP), and Western Dvina (DG, DN, IN) basins show close genetic affinities (Figure S1C). Notably, despite sharing the same drainage system, populations from the Upper Danube (e.g., SD, SN; MU, TK) and Middle Danube (e.g., SM, CS, MR) exhibit differentiation in their genetic constitution and show closer genetic relatedness to populations from the Neman (JZ, SP) and Western Dvina (DG, DN, IN) basins (Figure S1C). These results suggest that while within‐individual variation is substantial, geographic proximity and drainage system affiliation significantly influence the nuclear genetic structure of *U. crassus* populations.

Genetic differentiation among populations (pairwise *F*_ST_) ranged from −0.004 (TM/SM) to 0.305 (AS/SF and BO/SN) (Table S3). A clear pattern of genetic differentiation was observed between northern and southern German populations, with *U. crassus* populations in the north exhibiting high genetic differentiation (*F*_ST_ > 0.25) from *U. nanus* populations in the south. In contrast, populations of the species *U. crassus* , inhabiting the geographically distinct Baltic region of Neman and Dvina (SP, DG, IN), exhibited remarkably minimal to no genetic subdivision relative to Balkan populations from the Middle Danube (SM, TM). This striking lack of genetic differentiation across such a considerable range, all within *U. crassus* , strongly indicates a high degree of past gene flow that has maintained genetic homogeneity despite the species' colonization of these separate regions. Within the Balkan populations from the Middle Danube, genetic differentiation between *U. crassus* and *U. nanus* populations was generally low, except for the geographically isolated population TC, which exhibited high divergence. High genetic differentiation was also characteristic of populations located in the Upper Danube in the Alpine foreland (e.g., SF, EB, SN, and SZ). This pattern is indicative of genetic isolation at the peripheral range of the thick‐shelled river mussel in this region. Populations from the Meuse and Rhine systems (HE, LE, SU, XO, NI, GG, AB, and SZ) generally displayed low to moderate genetic differentiation. Interestingly, the sympatric populations GH and ME from the Scheldt and Meuse system, comprising both *U. crassus* and *U. nanus* , exhibited low genetic divergence to northern *U. crassus* populations and *U. nanus* populations from the Upper Danube. Furthermore, the sympatric populations from the Rhine system (AI) and from the Upper Danube (RG) showed low levels of differentiation compared to northern *U. crassus* populations and, at the same time, to *U. nanus* populations from the Meuse and Rhine system (e.g., HE, LE, SE, and XO). These findings suggest the presence of a secondary contact zone in the Scheldt‐Meuse‐Rhine region, characterized by ongoing or historical gene flow between species.

**FIGURE 3:**
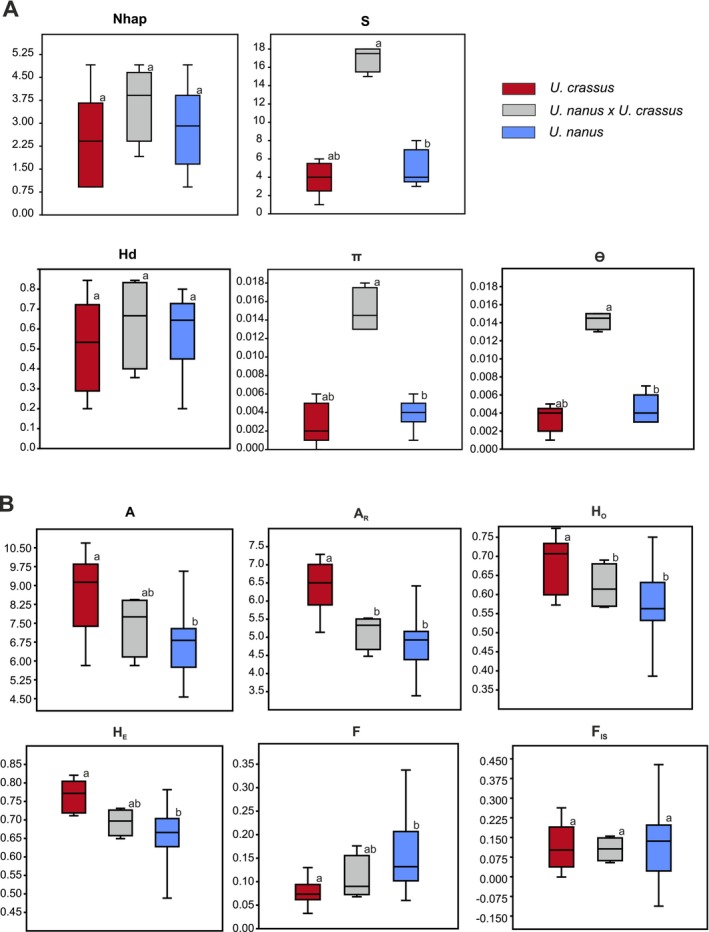
Boxplots of genetic diversity parameters between *U. nanus*, *U. crassus* , and *U. nanus* / *U. crassus* populations derived from the COI gene sequence analysis (A) with number of haplotypes (*N*_hap_), number of polymorphic sites (*S*), haplotype gene diversity (*H*_d_), nucleotide diversity (*π*), and Watterson's estimator of theta (*ϴ*) and derived from microsatellite analysis (B) with average number of alleles per locus (*A*), allelic richness (*A*_R_), observed and expected heterozygosity (*H*_O_ and *H*_E_), probability of allelic coancestry (*F*), and inbreeding coefficient (*F*_IS_). Significant differences are shown by different letters (a,b).

The average allele number per locus (*A*) was 7.30 and varied from a minimum value of *A* = 4.63 in population SZ to a maximum value of *A* = 10.75 in the northernmost population DG (Table 2, Figure 3B). Allelic richness standardized to the minimum sample size (*A*_R_) over all populations was *A*_R_ = 5.29. Lowest values of *A*_R_ were found in populations EB (*A*_R_ = 3.42) and SZ (*A*_R_ = 3.52), whereas highest values of *A*_R_ were found in population CS from the Middle Danube (*A*_R_ = 7.32). In general, *A*_R_ was found to be higher in populations from Neman and the Middle Danube, except for population TC, where the allelic richness was reduced compared to populations from the same drainage system. In contrast, populations from the Upper Danube and Scheldt–Meuse–Rhine system showed moderate *A*_R_ values below or around the average. The average expected heterozygosity and observed heterozygosity over all sixty populations were *H*_E_ = 0.703 and *H*_O_ = 0.621, respectively. Expected heterozygosities varied from *H*_E_ = 0.493 in population SN from the Upper Danube to *H*_E_ = 0.842 in population CS from the Middle Danube, while observed heterozygosities ranged from *H*_O_ = 0.390 in population AB from the Rhine to *H*_O_ = 0.795 in population TM from the Middle Danube. Low values of H_O_ were found in the peripheral populations EB (*H*_O_ = 0.446) and SN (*H*_O_ = 0.449) from the Upper Danube, indicating genetic isolation. The presence of private alleles in population EB (Uc77, 217 bp, 46.7%) suggests a unique genetic composition within this population. High values of *H*_O_ were detected in populations from the Elbe (IL and AL), Eider (BO), Kavlingeans (BR), Neman (JZ and SP), and Western Dvina (DG, DN, and IN) originating from the northern distribution limit. Although there are evident heterozygosity differences between populations, a spatial dependence of heterozygosity, for example in drainages, cannot be derived. However, populations assigned to the species *U. crassus* showed significantly higher values in the diversity parameters *H*_E_, *H*_O_, *A*, and *A*_R_ compared to populations assigned to *U. nanus* (*p* < 0.001), respectively (Table 2, Figure 3B).

**TABLE 2:** Microsatellite diversity indices of 60 thick‐shelled river mussel populations with population code (POPID), sample size (*N*), expected and observed heterozygosity (*H*_E_ and *H*_O_), average number of alleles per locus (*A*), mean allelic richness (*A*_R_), inbreeding coefficient (*F*_IS_), and proportion of common ancestors (*F*).

POPID	*N*	*H*_E_	*H*_O_	*A*	*A*_R_	*F*_IS_	*F*
GH	19	0.662	0.579	5.88	4.51	0.128	0.179
HA	25	0.720	0.705	6.88	4.97	0.021	0.131
HE	23	0.681	0.516	6.00	4.44	0.246	0.164
LE	25	0.675	0.540	7.50	5.10	0.204	0.109
ME	25	0.696	0.590	7.25	5.26	0.155	0.095
OU	25	0.726	0.610	8.00	5.55	0.162	0.061
SE	25	0.738	0.645	7.25	5.51	0.128	0.102
RU	20	0.714	0.575	6.75	5.10	0.198	0.111
LA	25	0.572	0.495	5.00	3.80	0.137	0.273
SU	23	0.721	0.647	7.00	5.21	0.105	0.117
UR	25	0.646	0.605	6.25	4.65	0.065	0.093
XO	25	0.700	0.705	7.13	4.95	−0.008	0.089
XS	24	0.741	0.630	7.13	5.41	0.153	0.086
NI	25	0.696	0.575	6.88	5.10	0.177	0.100
GG	29	0.669	0.526	7.50	5.02	0.217	0.129
AI	30	0.728	0.667	8.38	5.47	0.085	0.069
AB	30	0.676	0.390	7.25	5.04	0.428	0.134
SZ	30	0.575	0.599	4.63	3.52	−0.041	0.303
WL	30	0.636	0.575	5.63	4.29	0.098	0.246
GB	30	0.657	0.550	6.88	4.75	0.165	0.180
NB	30	0.710	0.704	6.88	5.14	0.008	0.134
EB	30	0.535	0.446	4.63	3.42	0.169	0.282
SF	30	0.551	0.575	4.75	3.63	−0.045	0.343
DM	30	0.648	0.646	7.38	4.86	0.004	0.105
BD	30	0.597	0.663	5.63	4.04	−0.112	0.238
MW	30	0.637	0.688	5.75	4.30	−0.081	0.285
SD	24	0.613	0.510	6.00	4.41	0.171	0.121
SL	30	0.713	0.565	7.13	5.14	0.210	0.132
ST	30	0.704	0.621	7.88	5.11	0.120	0.135
TK	30	0.650	0.663	6.50	4.72	−0.020	0.140
GL	30	0.678	0.546	7.75	5.27	0.195	0.084
KB	30	0.743	0.771	7.13	5.15	−0.038	0.148
KL	30	0.716	0.621	7.50	5.25	0.135	0.136
KU	30	0.669	0.567	6.63	4.94	0.156	0.134
MU	30	0.573	0.521	5.50	3.97	0.092	0.207
SN	22	0.493	0.449	4.75	3.72	0.092	0.238
FO	12	0.688	0.542	5.13	4.58	0.221	0.253
NA	30	0.724	0.571	9.63	6.03	0.214	0.074
PF	26	0.666	0.649	6.38	4.82	0.026	0.158
RG	30	0.748	0.708	8.50	5.56	0.054	0.088
CS	25	0.842	0.705	10.38	7.32	0.166	0.075
MR	25	0.789	0.585	9.25	6.53	0.263	0.097
TM	25	0.833	0.795	9.25	7.06	0.047	0.050
NR	25	0.730	0.545	8.50	5.95	0.258	0.101
SM	25	0.832	0.755	10.00	7.15	0.094	0.033
TC	25	0.683	0.570	6.38	4.87	0.168	0.211
SC	25	0.728	0.655	7.25	5.23	0.102	0.122
SR	24	0.801	0.573	8.50	6.45	0.289	0.103
CB	20	0.727	0.588	8.50	6.04	0.196	0.091
IL	29	0.782	0.749	9.63	6.72	0.043	0.074
BL	18	0.727	0.598	7.38	5.88	0.174	0.125
AL	12	0.815	0.729	7.38	6.54	0.109	0.088
AS	16	0.734	0.595	7.63	5.89	0.196	0.102
BO	13	0.738	0.738	5.88	5.17	−0.001	0.069
BR	18	0.733	0.722	7.13	5.75	0.016	0.132
JZ	25	0.792	0.764	9.13	6.24	0.036	0.066
SP	27	0.772	0.755	9.38	6.34	0.024	0.087
DG	31	0.828	0.750	10.75	7.18	0.095	0.065
DN	30	0.815	0.658	10.50	6.99	0.195	0.058
IN	16	0.808	0.711	8.63	6.68	0.123	0.062
Overall	1531	0.703	0.621	7.298	5.295	0.116	0.134

Likelihood tests employing the 2MOD program favored a gene‐flow‐drift model over a drift‐alone model (*P*[gene flow] = 1.00, Bayes factor = 8845), yielding an average *F*‐value across all populations of *F* = 0.134. However, some peripheral populations, such as SZ, SF, EB, SC, LA, and TC, showed high values of allelic coancestry (*F* > 0.2), suggesting genetic isolation due to isolation by geographic distance. Conversely, northern and southeastern populations displayed low levels of allelic coancestry (*F* < 0.1), indicating substantial historical and/or contemporary gene flow between them, except for the geographically isolated populations from the Middle Danube (TC and SC) originating from the inner Carpathian arc. Inbreeding coefficients ranged from *F*_IS_ = −0.112 in the Danubian population BD to *F*_IS_ = 0.428 in the Danubian population AB. Negative inbreeding coefficients were detected in eight out of 60 populations (BO, XO, SZ, SZ, SF, BD, MW, TK, and KB), which may indicate spatial Wahlund or founder effects. Surprisingly, the sympatric population NR ( *U. nanus* × *U. vicarius* ) exhibited a high inbreeding coefficient. No significant difference in inbreeding coefficients was observed between populations from either *U. crassus* , *U. nanus* , or *U. crassus* × *U. nanus* (Mann–Whitney‐*U*‐Test, *p* = 0.76, *p* = 0.96, *p* = 0.51).

Analysis of microsatellite data using BOTTLENECK 1.2.02 revealed evidence of recent population bottlenecks in 8 out of 60 populations (LE, ME, GG, DM, SD, ST, GL, SN, and CB), as indicated by significant heterozygosity excess. However, all populations showed a normal L‐shaped distribution of allele frequencies, suggesting an absence of recent bottleneck events. This apparent discrepancy may be attributed to the limitations of the Wilcoxon signed‐rank test employed in BOTTLENECK, which may be prone to false positives when population sizes are small and highly variable. Alternatively, the observed heterozygosity excess could reflect other factors, such as recent admixture or balancing selection, rather than a recent bottleneck.

### 3.3 Shell Morphology

**FIGURE 4:**
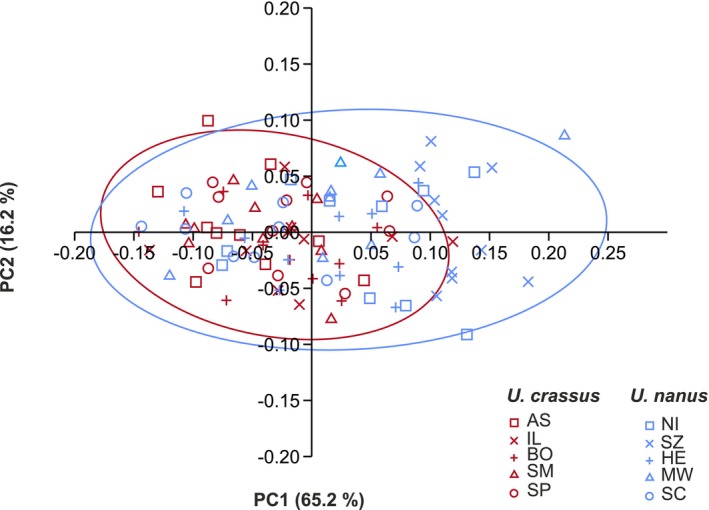
Scatterplot of the principal component analysis (PCA) displaying the first two principal components (PC1 and PC2) on 18 Fourier coefficients of 100 analyzed individuals comprising five populations from the species *U. crassus* and five populations from the species *U. nanus* . Colors correspond to the species, and color lines show 95% confidence ellipses. Population codes (POPID) are according to Table S1. Shell outlines of extreme morphotypes are shown on the four sides of the scatter plot.

PCA based on 18 Fourier coefficients was used to analyze shell shape variation (Figure 4). The first two principal components (PCs), PC1 and PC2, explained 65.2% and 16.2% of the total variation, respectively. PC1 primarily reflected variation in sagittal shell size, while PC2 captured variation in shell height. Tukey's post hoc pairwise comparisons showed that PC1 values differed significantly between *U. crassus* and *U. nanus* (*p* < 0.001), but PC2 values did not (*p* = 0.565). The two species were largely separated along PC1, indicating a clear difference in sagittal size. *U. nanus* exhibited greater overall shape variation than *U. crassus* , as evidenced by its wider spread across the PCA plot.

Despite the clear separation of species along PC1, LDA revealed that shell shape alone could not reliably assign specimens to their correct species. Only 62% of individuals were correctly assigned to their species classification using jackknifed cross‐validation. The proportions of correct assignments were low for both *U. crassus* (32%) and *U. nanus* (30%). Similarly, shell shape could not reliably assign specimens to their population of origin, with only 28% of specimens correctly assigned using jackknifed cross‐validation. Assignment rates at the population level were generally low (10%–40%), except for population SZ, which had a high assignment rate (> 80%). Individuals from population SZ showed a characteristic kidney‐shaped shell outline, which likely contributed to their higher assignment accuracy.

### 3.4 Reconstruction of Postglacial Colonization Routes

**FIGURE 5:**
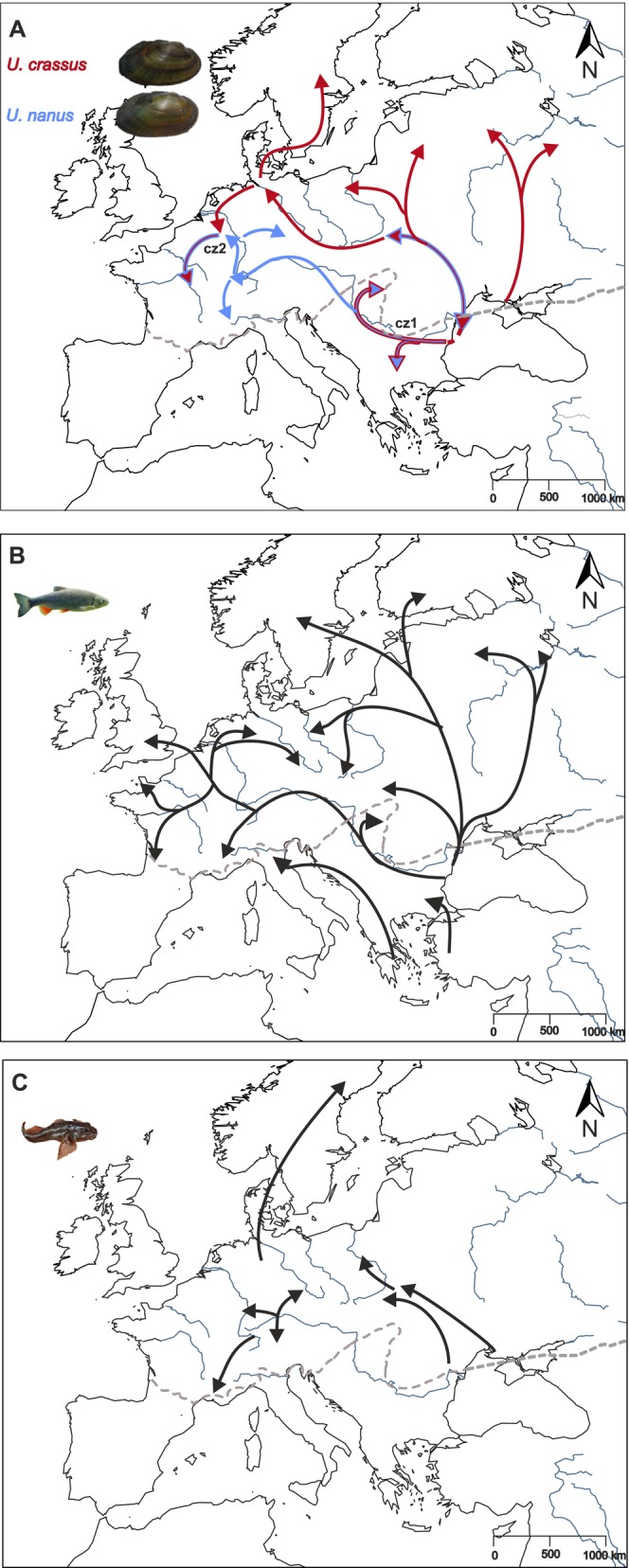
Reconstruction of possible postglacial colonization routes of (A) *U. crassus* (red arrows) and *U. nanus* (blue arrows); cz1 = secondary contact zone in the lower Danube basin and cz2 = secondary contact zone in the Scheldt‐Meuse‐Rhine system. (B) The European chub (*S. cephalus*) based on Hewitt 2011, and (C) The European bullhead ( *C. gobio* ) based on (Englbrecht et al. 2000). The dotted gray line indicates the approximate limit of the permafrost boundary during the last glacial maximum based on (Vandenberghe et al. 2014).

The general scenario of postglacial colonization by the thick‐shelled river mussel in Europe comprises two main routes originating from multiple southern LGM (Last Glacial Maximum) refugia around the Black Sea region (Figure 5A): (i) the Western route along the Danube river axis and expansion to the Rhine system outgoing from southern tributaries of the Danube, and (ii) the Eastern route starting in the periphery of the Black Sea and expansion northwards along the Dnieper‐Dniester river axes to the Baltic Sea basins. These routes converged in the Scheldt‐Meuse‐Rhine region, forming a secondary contact zone where recent admixture between species ( *U. crassus* × *U. nanus* ) occurred after retreating ice sheets. Unexpectedly, admixture between these species is also apparent in the southern Danubian LGM refuge, suggesting evidence of an older Black Sea–Caspian Sea divergence between *U. nanus* and *U. crassus* . The European chub (*S. cephalus*), a temperate freshwater fish, likely experienced significant range contraction during the LGM, with potential eradication from much of Europe (Durand et al. 1999; Sanjur et al. 2003). Several authors propose survival in southern refugia, including areas south of the Danube and the peripheries of the Black and Caspian Seas (Figure 5B; Hewitt 2004; Costedoat and Gilles 2009; Seifertová et al. 2012). Seifertová et al. (2012) further suggest a rapid northward expansion from these southern refuges. For the Danubian (western) lineage of the European chub, a two‐step recolonization process is proposed. During the Riss‐Würm interglacial period, this lineage likely expanded from the Danube to western Europe (Rhine‐Rhone‐Loire drainages) (Durand et al. 1999). This scenario suggests an initial westward expansion followed by a subsequent northward recolonization after the LGM. In contrast to the European chub, the cold‐adapted European bullhead ( *C. gobio* ) likely persisted in multiple refugia during glacial periods, including Central Europe (Figure 5C; Englbrecht et al. 2000). Its initial colonization of Central Europe occurred along the lower Danube in the Pliocene. Subsequently, one of these populations reached the northern coast, facilitating the further colonization of western Europe along the coastline. Eastern Europe was colonized independently during the Pleistocene, while Fennoscandia was recolonized in the Holocene following the last glacial retreat.

## 4 Discussion

This study revealed a significant discordance between mitochondrial and nuclear genetic structuring within the currently recognized species *U. crassus* and *U. nanus* . While mitochondrial DNA displayed distinct species differentiation, this pattern was not reflected in the nuclear DNA. This observation aligns with the well‐established phenomenon of mito‐nuclear discordance (Funk and Omland 2003; Toews and Brelsford 2012), a common occurrence in bivalve mollusks (Chong et al. 2016; Lee et al. 2021; Formaggioni et al. 2022; Liza et al. 2024). Introgression from phylogenetically divergent populations is frequently invoked as a primary mechanism driving such discordance (Formaggioni et al. 2022). While drainage patterns and mitochondrial species provide partial insights into the observed genetic structure, the findings in this study suggest that the colonization history of the thick‐shelled river mussel and their host fish plays a substantial role. The distinct separation of mitochondrial species provides evidence for an older Black Sea–Caspian Sea divergence between *U. nanus* and *U. crassus* , aligning with the emerging paradigm of a Black Sea–Caspian Sea division as a significant driver of genetic differentiation in aquatic organisms (Hewitt 2004; Nesbø et al. 1999). Notably, no evidence for recent hybridization events between *U. crassus* and either *U. nanus* or *U. vicarius* was found. The absence of F1 hybrids strongly suggests that the primary hybridization event is not ongoing. Instead, the observed presence of F2 and subsequent generations indicates that gene flow occurred historically. This pattern implies that genetic material was transferred between distinct lineages in the past and has since undergone extensive recombination and backcrossing over multiple generations. Therefore, the observed mito‐nuclear discordance is likely a consequence of ancient introgression, potentially driven by past hydrological and climatic fluctuations (Hewitt 2011).

The admixture and introgression of distinct *U. crassus* and *U. nanus* species within the southern Danubian refuge are most likely attributed to the hydrological history of the Black Sea with highly dynamic salinity fluctuations. The Black Sea has undergone multiple freshwater phases over the past 670,000 years (Badertscher et al. 2011, Degens and Ross 1972), facilitating the dispersal of freshwater fishes. These fishes likely acted as vectors for mussel larvae, enabling their dispersal and contributing to the sympatric distribution observed today. This phenomenon of fish dispersal during freshwater phases of the Black Sea has been documented for numerous aquatic species (Economidis and Banarescu 1991; Kotlík et al. 2004), highlighting its role in shaping the region's biodiversity. More precisely, the species *U. nanus* contributed predominantly to the dispersion into the Upper Danube and Scheldt–Meuse–Rhine rivers along the Western route. The recolonization via the Western route might have happened in a one‐step scenario or a two‐step scenario as suggested for the European chub (Durand et al. 1999). During the LGM, the Rhine glacier significantly altered the landscape, and glacial meltwater likely created temporary connections between different river systems. As the glaciers retreated, some of these connections may have persisted for some time, allowing fish to move between the Danube and Rhine watersheds (Behrmann‐Godel et al. 2004; Gouskov and Vorburger 2016). During glacial periods, Danubian tributaries changed their flow direction to the Rhine system through geological stream capture and shifting watersheds (Hötzl 1996; Yanites et al. 2013).

In contrast to the multi‐step expansion of *U. nanus* , the postglacial colonization of *U. crassus* appears to have followed a single, rapid northward route from Black Sea refugia to the Baltic region via the Dnieper or Dniester rivers (Figure 5A). This expansion likely occurred during the freshwater Ancylus Lake phase around 10,000 BP (Fredén 1967; Jensen et al. 1999), facilitated by dispersal via cold‐adapted fish species, such as the European bullhead ( *C. gobio* ), known to have colonized the Baltic during this period (Kontula and Väinölä 2004). The close host‐fish affiliation of *U. crassus* with the European bullhead in this region (Schneider et al. 2017) further supports this hypothesis. Subsequently, *U. crassus* expanded westward to the Atlantic regions via the Channel River, a paleo river connecting major northwestern European rivers (Ménot et al. 2006; Boswell et al. 2019). Again, the European bullhead, the primary host fish in this region (Lamand et al. 2016), likely played a crucial role in the dispersal of the thick‐shelled river mussel. This westward expansion led to the convergence of the eastern and western colonization routes in the Scheldt–Meuse–Rhine region, forming a secondary contact zone where admixture between *U. crassus* and *U. nanus* species occurred. The limited presence of *U. crassus* in certain Upper Danube tributaries may be attributed to anthropogenic influences, such as human‐mediated translocation of glochidia‐infested fish stocks or the introduction of *U. crassus* from the Rhine system via the artificial Rhine‐Main‐Danube Canal.

The significant difference in nuclear genetic diversity between *U. crassus* and *U. nanus* , particularly in allelic richness and heterozygosity, is a key finding of this study with important implications for conservation. Contrary to the central‐peripheral hypothesis, *U. crassus* exhibits high nuclear genetic diversity, whereas *U. nanus* shows lower diversity. This pattern aligns with observations for the thick‐shelled river mussel in Feind et al. (2018). While lower genetic diversity might suggest a higher extinction risk for *U. nanus* , there is no evidence of reduced fitness, such as inbreeding depression. The low allelic richness observed in some *U. nanus* populations, including outbreeding populations (e.g., SZ, SF, BD, MW), can be attributed to founder effects and genetic drift rather than inbreeding, as also evident in other freshwater mussel species in Europe (e.g., Geist et al. 2025). Alleles get lost randomly due to drift effects, especially in small populations, further reducing genetic diversity. In addition, habitat fragmentation, for example due to declines in connectivity and habitat quality (Dobler et al. 2024), can lead to smaller, isolated populations, increasing the impact of genetic drift and potentially leading to further loss of diversity. Therefore, the lower genetic diversity in *U. nanus* likely reflects demographic history and genetic drift rather than an inherently lower adaptive potential.

Shell morphology alone is unreliable to distinguish between *U. crassus* and *U. nanus* , as demonstrated for the *U. crassus* complex (this study, Lopes‐Lima, Geist, et al. 2024) and other freshwater bivalves (Beggel et al. 2015). Cryptic species with similar shell shapes hinder accurate species identification, and potential interspecific introgression can lead to intermediate morphologies, further complicating species delimitation. Shell morphology in aquatic mollusks is often influenced by environmental factors such as water flow, substrate type, and predator pressure, leading to ecophenotypic plasticity, where populations develop different shapes in response to local conditions. Understanding this variation can provide insights into the habitat‐specific adaptations of *U. nanus* and *U. crassus* populations and the environmental stressors they experience. For conservation, identifying such morphological differentiation, even if not indicative of distinct species, can highlight ecologically distinct units that may warrant specific management strategies. The high degree of shell shape variation observed in *U. nanus* may reflect greater ecological or environmental heterogeneity experienced by this species. This variation could be associated with adaptations to different habitats or ecological niches occupied by each population or individual, as shell shape is known to be influenced by environmental factors such as microhabitat conditions (Bujas et al. 2024; Zając, Zając, and Ćmiel 2018).

Contemporary biodiversity conservation strategies predominantly emphasize geographic regions, ecosystems, ecological communities, and species‐level entities, often with comparatively limited consideration of intraspecific genetic diversity and the evolutionary continuum that spans populations to species (Coates et al. 2018). Conservation strategies must account for the distinct genetic diversity levels and the remarkable inter‐individual genetic variation observed. This necessitates incorporating genetic data into conservation assessments, such as the IUCN Red List, to ensure the preservation of both neutral and adaptive genetic diversity. The recent taxonomic split of the *U. crassus* complex into distinct species, as supported by this study, means that previous Red List evaluations, based on the broader *U. crassus* agg. complex, may have masked the true conservation status of individual cryptic species. This information is vital for delineating Evolutionary Significant Units (ESUs), ensuring that conservation efforts protect the full breadth of genetic diversity within the complex, rather than treating them as a single, undifferentiated species. A reassessment is crucial to ensure that the distinct demographic histories, genetic vulnerabilities, and specific threats faced by *U. nanus* and *U. crassus* are accurately reflected in their conservation categorization. While *U. nanus* exhibits lower neutral diversity, it may still possess sufficient adaptive potential to persist in its current environment. However, lower genetic diversity may limit its ability to adapt to future environmental changes and therefore account for a higher risk of extinction. Therefore, conservation efforts should prioritize maintaining both neutral and adaptive genetic diversity in both species to ensure their long‐term survival. Conservation actions should prioritize maintaining and enhancing genetic diversity in *U. nanus* through habitat restoration, connectivity improvements, and potentially translocation programs to mitigate the effects of genetic drift and fragmentation.

The current genetic patterns of *U. nanus* and *U. crassus* appear to be strongly influenced by their respective host‐fish associations and their past colonization history. *U. nanus* exhibits a closer relationship with temperate fish species, such as the European chub, while *U. crassus* appears more reliant on cold‐adapted host fish, for example the European bullhead. This differential host‐fish dependency, together with their colonization history, likely played a significant role in shaping the distinct distribution patterns observed for these two species. To clarify these host‐fish preferences for *U. crassus* and *U. nanus* , controlled (laboratory) cross‐infestation experiments are recommended (Taeubert et al. 2013). These experiments would involve exposing both mussel species to temperate and cold‐adapted host‐fish species from different regions and assessing their respective infectivity and survival rates.

Finally, DNA barcoding utilizing the COI gene sequence is proposed as a reliable method for species identification, superseding traditional shell shape analysis, which can be unreliable due to phenotypic plasticity or introgression. In conclusion, while prior research has established key host‐fish associations for the thick‐shelled river mussel complex, these findings predominantly relied on methods that did not differentiate between the closely related species of *U. crassus* and *U. nanus* . Therefore, future investigations explicitly incorporating the species of the mussel in analyses of host‐fish interactions are crucial for elucidating potential species‐specific host preferences within this complex.

## Author Contributions

**Sarah Egg:** conceptualization (equal), data curation (lead), formal analysis (lead), investigation (lead), visualization (lead), writing – original draft (lead). **Manuel Lopes‐Lima:** conceptualization (equal), formal analysis (supporting), investigation (supporting), methodology (supporting), validation (supporting), writing – review and editing (supporting). **Helmut Bayerl:** investigation (supporting), writing – review and editing (supporting). **Elsa Froufe:** data curation (supporting), investigation (supporting), writing – review and editing (supporting). **Bernhard C. Stoeckle:** investigation (supporting), methodology (supporting), writing – review and editing (supporting). **Ralph Kuehn:** conceptualization (equal), formal analysis (equal), methodology (equal), software (equal), supervision (supporting), validation (equal), writing – review and editing (supporting). **Juergen Geist:** conceptualization (equal), funding acquisition (lead), methodology (equal), resources (equal), supervision (lead), validation (equal), writing – review and editing (equal).

## Conflicts of Interest

The authors declare no conflicts of interest.

## Supporting information


**Data S1:** ece372113‐sup‐0002‐DataS1.zip.

## Data Availability

Individual genotype data and unique haplotype data are available on DataDryad https://doi.org/10.5061/dryad.3tx95x6ts.
