# Supplementary material for: The Impact of Glacial Disturbance History Upon the Genetic Diversity of Unio crassus and Unio nanus in Europe and Implications for Conservation
Source: Ecol Evol. 2025 Sep 6;15(9):e72113. doi: 10.1002/ece3.72113 (PMC12413660; doi:10.1002/ece3.72113)
Supplement: Supplementary file 1 — Data S1: ece372113‐sup‐0002‐DataS1.zip. [file ECE3-15-e72113-s001.zip › Supporting Information for online publication.docx]

**Supplemental Information for:**

**The Impact of Glacial Disturbance History Upon the Genetic Diversity of *Unio crassus* and *Unio nanus* in Europe and Implications for Conservation**

Sarah Egg, Manuel Lopes-Lima, Helmut Bayerl, Elsa Froufe, Bernhard Christian Stoeckle, Ralph Kuehn, Juergen Geist

**Table of Contents:**

| **Figure S1 Figure Legend** | SuppInfo_Figure_S1.pdf |
| --- | --- |
| **Table S1 Table Legend** | SuppInfo_Table_S1.xlsx |
| **Table S2 Table Legend** | SuppInfo_Table_S2.xlsx |
| **Table S3 Table Legend** | SuppInfo_Table_S3.xlsx |

**Figure S1.** Discriminant analysis of principal components (DAPC) based on microsatellite data of 1531 individuals using 60 principal components and two discriminant functions. Similar colors characterize similar genetic constitution. A: Genetic clustering of individuals represented as dots using the mean population color based on the DAPC. Populations are indicated within 95 % inertia ellipses. B: Individual genetic constitution based on the DAPC sorted by population and drainage system. C: Geographical distribution with genetic characterization of populations based on the DAPC.

**Table S1.** Sampling characteristics of 60 thick shelled river mussel populations: population code (POPID), species, river name, drainage system, country, geographic coordinates (WGS84), sample material (TS: tissue, HL: hemolymph), reference to used microsatellite data, sample size used for microsatellite analysis (N_Ms_), for cytochrome c oxidase subunit I analysis (N_COI_) and for shell outline analysis (N_EFD_).

**Table S2.** AMOVA table of molecular variation from microsatellite data among species, among drainage systems, among and within populations using ARLEQUIN 3.5.

**Table S3.** Genetic differentiation between populations based on microsatellite data: pairwise F_ST_ estimates between populations (above diagonal), pairwise F_ST_ estimates between populations using ENA method (below diagonal), significant values are written in bold (*P* < 0.05 after Bonferroni correction).
