# Supplementary material for: The Impact of Glacial Disturbance History Upon the Genetic Diversity of Unio crassus and Unio nanus in Europe and Implications for Conservation
Source: Ecol Evol. 2025 Sep 6;15(9):e72113. doi: 10.1002/ece3.72113 (PMC12413660; doi:10.1002/ece3.72113)
Supplement: Supplementary file 1 — Data S1: ece372113‐sup‐0002‐DataS1.zip. [file ece372113-sup-0001-FigureS1.pdf › ece372113-sup-0001-FigureS1.pdf]

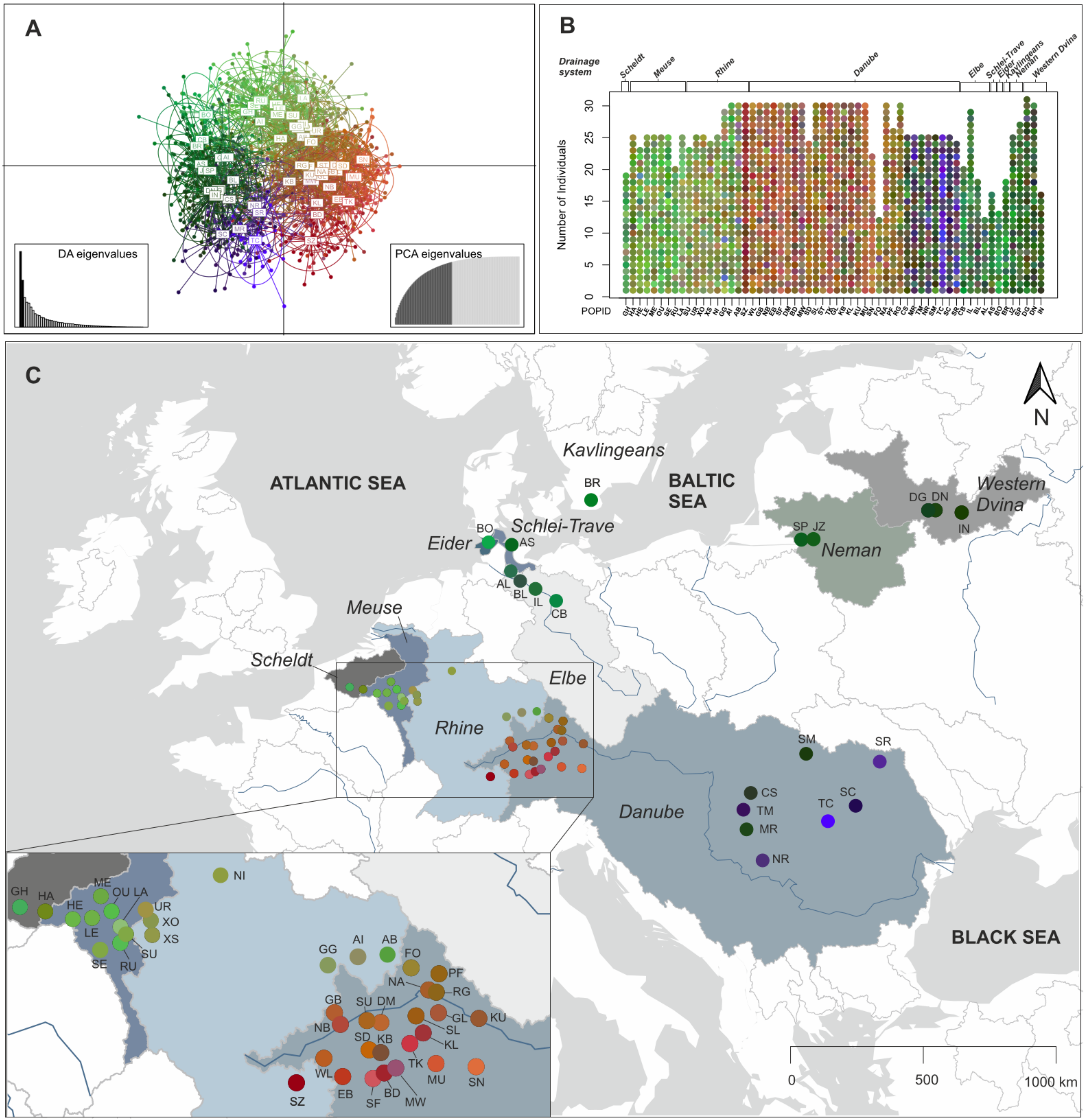
A
DA eigenvalues
PCA eigenvalues
B
Drainage system
Scheldt
Meuse
Rhine
Danube
Elbe
Schlei-Trave
Eider
Kavlingeans
Neman
Western Dvina
Number of Individuals
POPID
C
ATLANTIC SEA
BALTIC SEA
BLACK SEA
Kavlingeans
Schlei-Trave
Eider
Meuse
Scheldt
Elbe
Rhine
Danube
Neman
Western Dvina
N
0
500
1000 km
